# Prevalence of overuse of short-acting beta-2 agonists (SABA) and associated factors among patients with asthma in Germany

**DOI:** 10.1186/s12931-021-01701-3

**Published:** 2021-04-16

**Authors:** Heinrich Worth, Carl-Peter Criée, Claus F. Vogelmeier, Peter Kardos, Eva-Maria Becker, Karel Kostev, Ingo Mokros, Andrea Schneider

**Affiliations:** 1 Facharzt Forum Fürth, Bahnhofplatz 6, 90762 Fürth, Germany; 2 Evangelisches Krankenhaus Göttingen-Weende, Göttingen, Germany; 3 Department of Medicine, Pulmonary and Critical Care Medicine, University Medical Center Giessen and Marburg, Philipps-Universität Marburg, Marburg, Germany; 4 Lungenpraxis am Maingau Krankenhaus, Frankfurt am Main, Germany; 5 IQVIA, Frankfurt am Main, Germany; 6 AstraZeneca GmbH, Wedel, Germany

**Keywords:** Asthma, Short-acting beta agonists, Overuse, GINA, Risk factors, Germany

## Abstract

**Background:**

Overuse of short-acting beta-2 agonists (SABA), which do not treat the underlying inflammation of asthma, is linked to poor clinical outcomes such as increased exacerbation risk. This study, as part of the SABINA program, estimated the prevalence of SABA overuse and associated variables in outpatients in Germany.

**Methods:**

This retrospective study used anonymized electronic healthcare data from the Disease Analyzer database (IQVIA). A total of 15,640 patients aged ≥ 12 years with asthma who received ≥ 1 SABA prescription(s) between July 2017 and June 2018 in 924 general physician and 22 pneumologist (PN) practices were included. SABA overuse was defined as ≥ 3 prescribed inhalers (~ 200 puffs each) during the study period. The associations between SABA overuse and physician specialty, Global Initiative for Asthma (GINA) steps (based on asthma medications), age, sex, and inhaled corticosteroid (ICS)/long-acting beta agonist (LABA) use were estimated using multivariable regression for patients with probable moderate (GINA step 2) and probable severe (GINA steps 3–5) asthma.

**Results:**

Annually, 36% of all patients (GINA steps 1–5) in general and 38% in PN practices received ≥ 3 SABA inhalers. The risk of SABA overuse was 14% higher in patients treated by a general practitioner vs. a PN; 34% and 85% higher in GINA steps 4 and 5, respectively, vs. GINA step 3; and 40% higher in male vs. female patients.

**Conclusions:**

SABA overuse is prevalent among patients with asthma across all GINA steps in Germany, which may indicate suboptimal asthma control. Further studies are needed to investigate the reasons behind SABA overuse.

## Background

The lifetime prevalence of asthma in adults in Germany is approximately 6–9% [1, 2]. The prevalence varies considerably between federal states in Germany and is known to be higher in adult women than in men and in people with a low level of education [2]. Severe asthma is present, by definition, when adequate control of asthma cannot be achieved by high-dose treatment with inhaled corticosteroids (ICS) and additional controllers (inhaled long-acting beta-2 agonists [LABA], montelukast, and/or theophylline) and/or by oral corticosteroid treatment or if efficacy is lost when treatment is reduced [3].

One of the known problems in asthma patient care is the overuse of inhaled short-acting beta-2 agonists (SABA) [4, 5]. Overuse of reliever inhalers is a common problem in people with asthma, which was highlighted by the Medical Expenditure Panel Survey finding that 15% of the asthma population in the United States used more than one reliever inhaler per month [6]. Based on existing studies, it can be concluded that opinions on the duration of time needed to determine SABA overuse varies from daily and weekly doses to a monthly inhaler count. According to a Global Initiative for Asthma (GINA) report, the use of a reliever inhaler for symptoms more than twice per week in the past 4 weeks is classified as partly controlled asthma. If symptoms and activity limitation due to asthma are present nevertheless, it is classified as uncontrolled asthma [7].

The inclusion of reliever inhaler use in the assessment of asthma control in adults is based on the evidence that overuse of SABA medication is associated with poor symptom control [8], increased risk of exacerbations [9, 10], and death from asthma [11, 12].

Factors associated with inappropriate or excessive use of SABA include male sex, low socioeconomic status, and low continuity of care. However, knowledge of these factors is based on studies performed in countries other than Germany [13].

There are limited data on the prevalence of SABA overuse in Germany, and especially on the factors associated with it [4]. For example, Janson et al. investigated the prevalence of SABA overuse based on the number of canisters; however, these canisters could contain different number of puffs [4]. Given the large number of patients with asthma, the high prevalence of SABA overuse, and the substantial risk of complications associated with this overuse, it is important to use available German epidemiological databases to analyze SABA overuse, comparing different definitions within the same data source.

The **SABA** use **IN A**sthma (SABINA) program, which has been previously described [4], aims to describe asthma treatment prescription patterns, the extent of SABA overuse, and its impact on asthma-related clinical outcomes through a series of large observational cohort studies in different countries. This study, which is part of the SABINA program, aims to evaluate the prevalence of SABA overuse and factors associated with this overuse in German outpatient care.

## Methods

### Database

This study is based on data from the Disease Analyzer database (IQVIA), which compiles drug prescriptions, diagnoses, and basic medical and demographic data obtained directly and anonymously from computer systems used in the practices of general practitioners (GPs) and specialists. Diagnoses (International Classification of Diseases, 10th Revision [ICD-10]), prescriptions (Anatomical Therapeutic Chemical [ATC] classification system), and the quality of reported data were monitored by IQVIA based on multiple criteria (e.g., completeness of documentation, linkage between diagnoses and prescriptions) [14]. In Germany, the sampling methods used for the selection of physicians’ practices were appropriate for obtaining a representative database of general and specialized practices [14]. Finally, this database had already been used in studies focusing on asthma [15] and drug utilization [16, 17].

### Study population

**Fig. 1:**
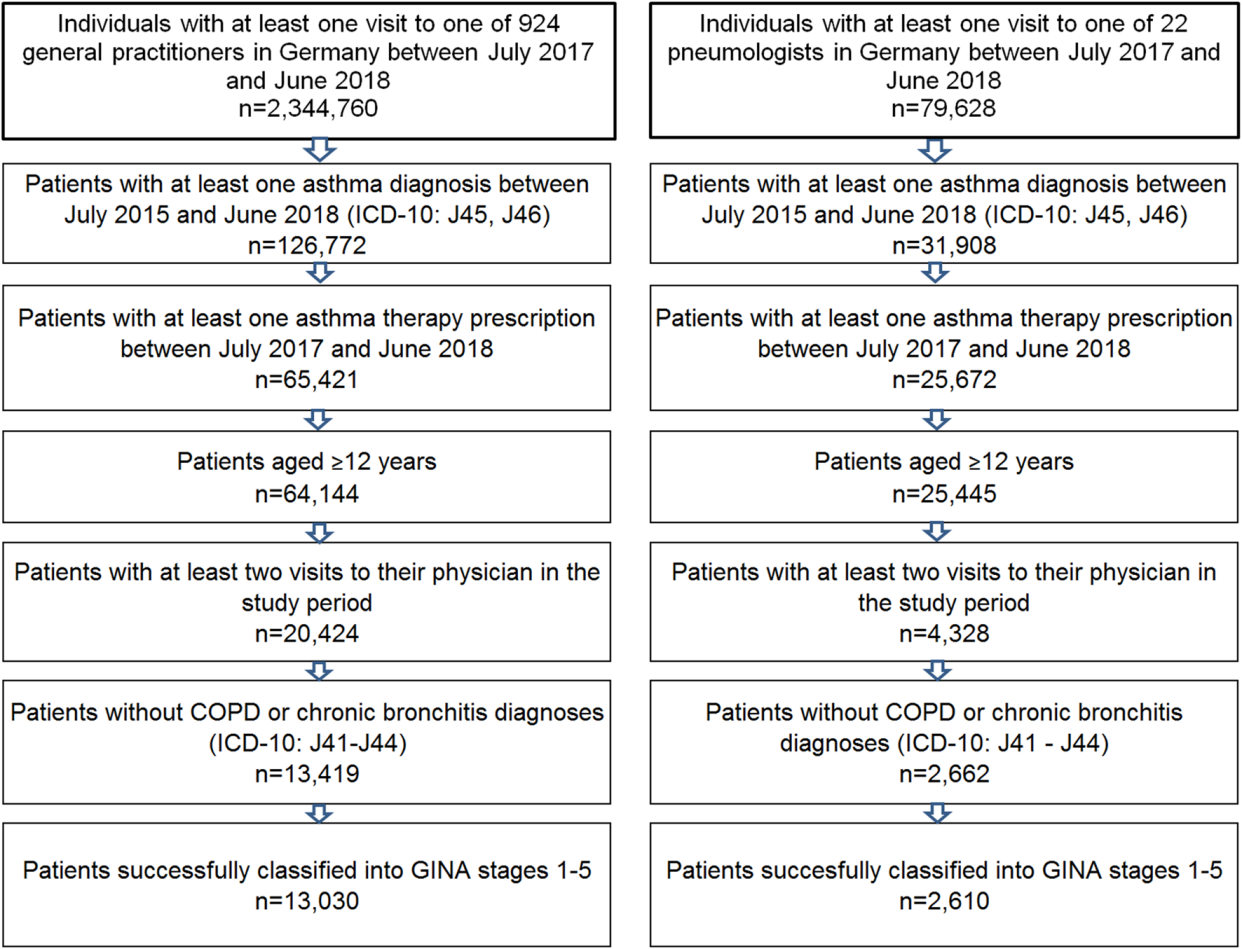
Selection of study patients. *COPD* chronic obstructive pulmonary disease, *GINA* Global Initiative for Asthma, *ICD-10* International Classification of Diseases, 10th Revision. A small percentage of patients received medications that could not be categorized into one of the five GINA stages

This cross-sectional study included outpatients aged ≥ 12 years with an asthma diagnosis (ICD-10: J45, J46) who had received ≥ 1 SABA (European Pharmaceutical Market Research Association [EphMRA] ATC: R03A4) prescription(s) between July 2017 and June 2018 in 924 general physician and 22 pneumologist (PN) practices. Furthermore, patients had to fulfill the observability criterion of having had at least two physician visits (not necessarily asthma-related) during the study period. Patients with chronic obstructive pulmonary disease in addition to their asthma diagnosis were excluded from the study (Fig. 1).

### Study outcome

The main outcome of this study was the prevalence of SABA overuse/increased use and assessment of its association with different factors. Based on GINA, SABA overuse was defined as ≥ 3 prescribed inhalers (~ 200 puffs each) during the study period. The term “overuse” was appropriated from the GINA report; however, it should be noted that in GINA step 5, the use of ≥ 3 prescribed inhalers is considered as increased use but not overuse. The prevalence of SABA overuse was estimated as the proportion of patients receiving ≥ 3 prescribed inhalers out of all patients with asthma included in the study. The prevalence was calculated for the total number of patients as well as stratified by age group, sex, GINA step, physician specialty, ICS with/without LABA (EphMRA ATC: R03A3, R03D1, R03F1) use, defined comorbidities (upper respiratory infections, diabetes mellitus, thyroid gland disorders, hypertension, lipid metabolism disorders, depression), co-therapies which are known to be associated with an increased risk for bronchospasm (non-steroidal anti-inflammatory drugs [NSAIDs] [ATC: M01A], aspirin [ATC: B01C1], and angiotensin-converting enzyme [ACE] inhibitors [ATC: C09A, C09B]) [18–20]. Moreover, the associations of these variables with SABA overuse were investigated.

### Statistical analyses

SABA overuse analyses were of a descriptive nature. The associations between SABA overuse and physician specialty, GINA step (based on prescribed asthma medications), age, and sex were estimated using multivariable regression. Co-diagnoses and co-therapies were also included in the model. ICS/LABA use was not included in the logistic regression because the proportion of patients with ICS/LABA use was a part of the GINA definition, and 95% of patients at GINA steps 3–5 were treated with ICS/LABA. *P* values < 0.05 were considered statistically significant. All analyses were carried out using SAS version 9.4 (SAS Institute, Cary, NC).

## Results

### Baseline characteristics of study patients

A total of 15,640 patients (13,030 GP patients and 2,610 PN patients) with ≥ 1 SABA prescription(s) during the study period were included in the study. The mean (standard deviation) age of patients was 49 (18) years for GP patients and 56 (16) years for PN patients. The proportion of female patients was 59% in general practices and 68% in PN practices, while 21% of GP patients and 49% of PN patients were classified as being at GINA treatment step 4 or 5 (Table 1).

**Table 1:** Baseline characteristics of asthma patients under general physician and pneumologist care in Germany

Variable	GPs	PNs
N	13,030	2610
GINA classes		
GINA 1	5426 (42)	99 (4)
GINA 2	841 (6)	165 (6)
GINA 3	4012 (31)	1065 (41)
GINA 4	2410 (18)	1064 (41)
GINA 5	341 (3)	217 (8)
Age (years)		
Mean (SD)	49 (18)	56 (16)
12 to < 18	244 (2)	29 (1)
18 to 65 years	10,330 (77)	1780 (68)
> 65 years	2456 (19)	801 (31)
Sex		
Female	7665 (59)	1770 (68)
Male	5364 (41)	840 (32)
ICS/LABA prescriptions		
Yes	7227 (55)	2461 (94)
No	5803 (45)	149 (6)

*GINA* Global Initiative for Asthma, *GP* general practitioner, *ICS* inhaled corticosteroid, *LABA* long-acting beta agonist, *PN* pneumologist, *SD* standard deviation

Data are presented as n (%) unless stated otherwise

### Prevalence of SABA overuse

**Fig. 2:**
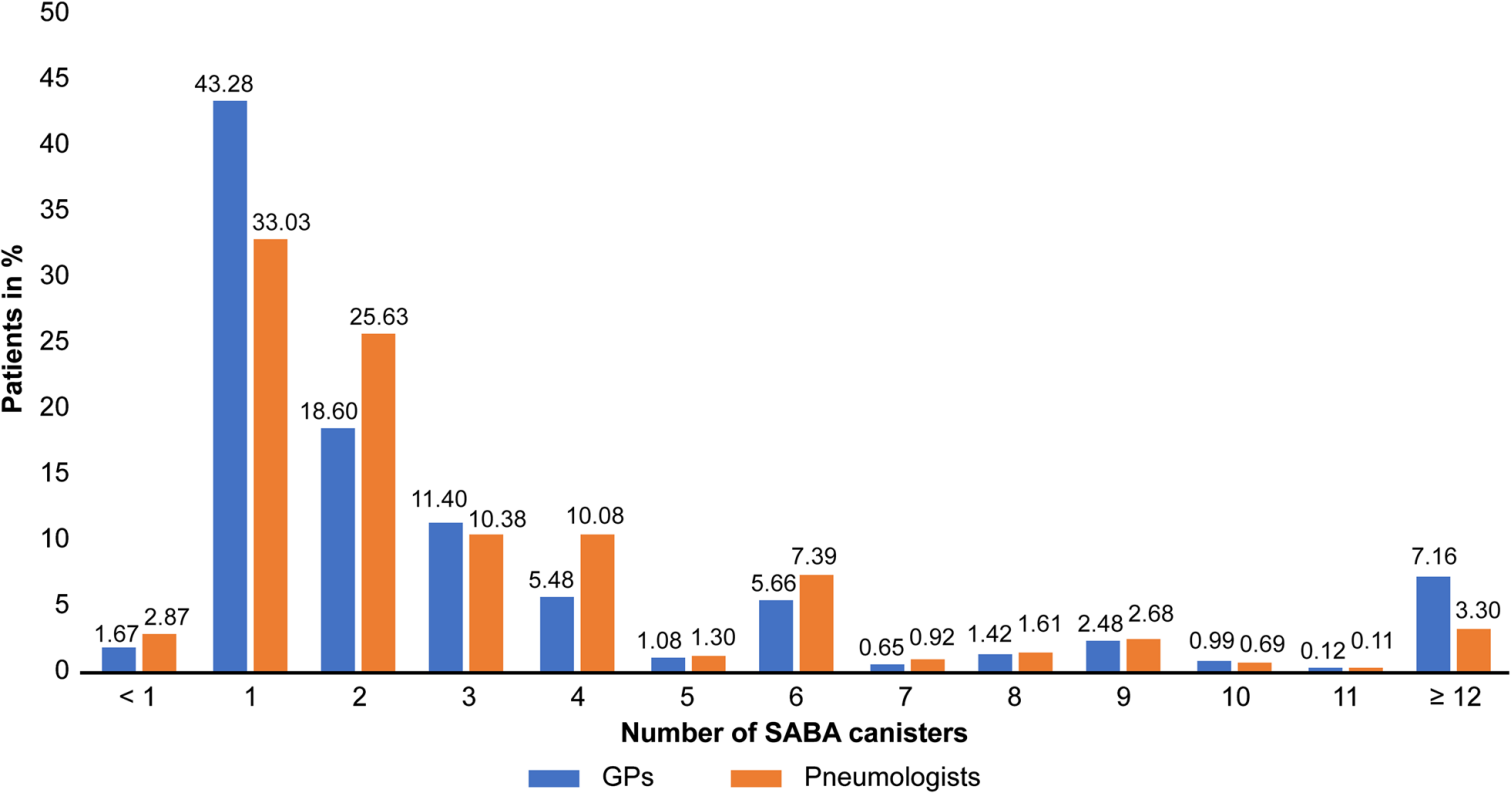
Number of SABA canisters prescribed to patients with asthma in general and pneumologist practices in Germany. *SABA* short-acting beta-2 agonist, *GP* general practitioner. To overcome variability in available SABA canister volumes and maintain consistency with the published threshold of SABA use, use of a canister was defined as 200 puffs by prescription. Consequently, if a patient received only one prescription for a canister containing < 200 puffs, they were classified as receiving < 1 canister

**Fig. 3:**
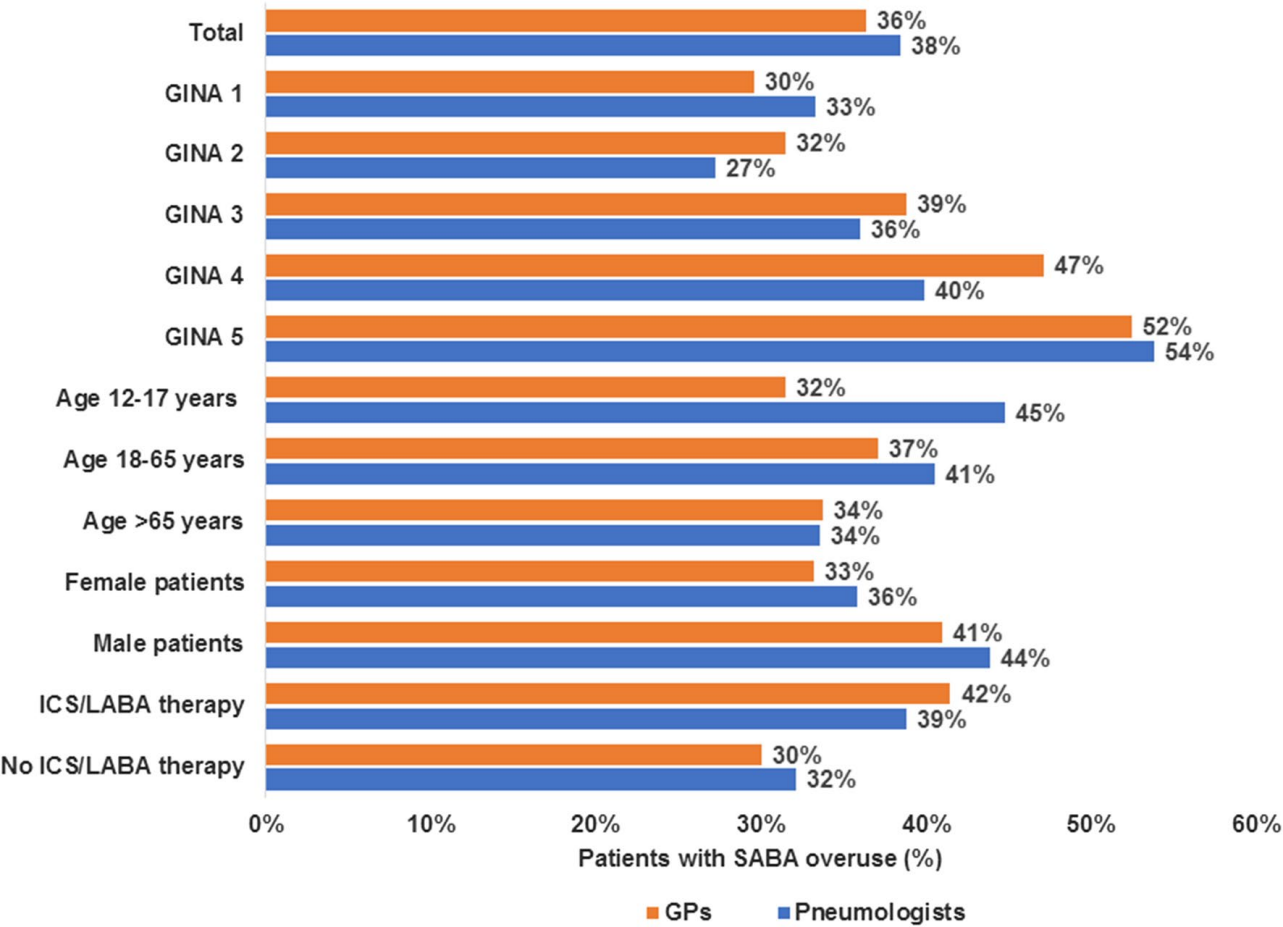
Prevalence of SABA overuse in patients treated in general and pneumologist practices in Germany. *GINA* Global Initiative for Asthma, *GP* general practitioner, *ICS* inhaled corticosteroid, *LABA* long-acting beta agonist, *SABA* short-acting beta-2 agonist

Annually, 36% of all GP patients (GINA steps 1–5) and 38% of all PN patients received ≥ 3 SABA inhalers. Over the same period, 7% of GP patients and 3% of PN patients received ≥ 12 inhalers (Fig. 2). The proportion of patients with SABA overuse increased with GINA step (52% seen by GPs and 54% seen by PNs). Moreover, this proportion was higher in men and in patients who received ICS/LABA therapy (Fig. 3).

### Variables associated with SABA overuse

**Fig. 4:**
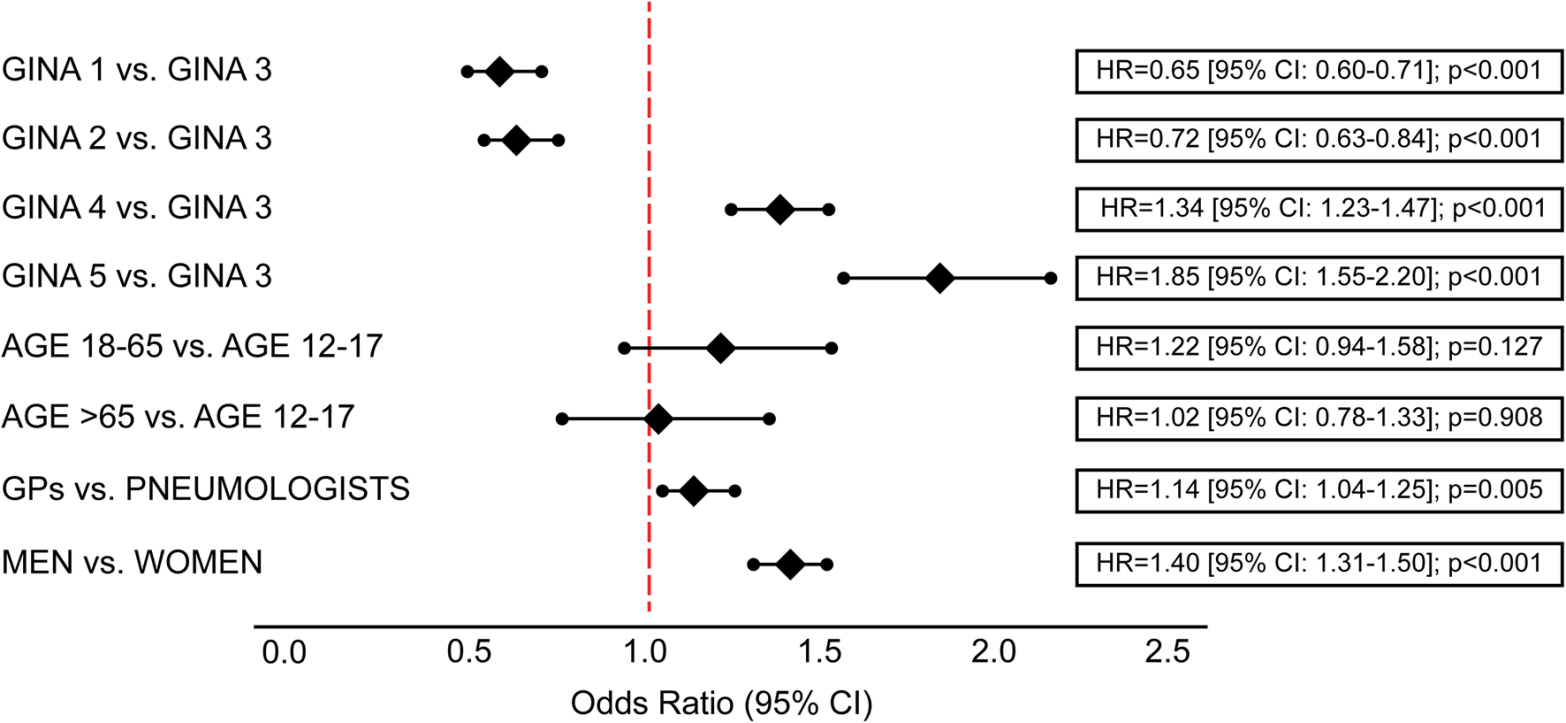
Variables associated with SABA overuse (multivariable logistic regression). *CI* confidence interval, *GINA* Global Initiative for Asthma, *HR* hazard ratio, *GP* general practitioner, *SABA* short-acting beta-2 agonist

Figure 4 displays the results of the multivariable logistic regression. The risk of SABA overuse/increased use was 14% higher in patients treated by a GP vs. a PN; 34% and 85% higher in GINA steps 4 and 5, respectively, vs. GINA step 3; and 40% higher in male vs. female patients. No significant effects were observed for other variables.

## Discussion

### Summary of main findings

This German retrospective study of 15,640 patients with asthma showed that the proportion of patients with SABA overuse/increased use was very high, especially among patients treated by GPs, patients classified as being at GINA step 4 or 5, and male patients.

### Prevalence of SABA overuse

In the multicountry study based on five European countries including Germany, Janson et al. investigated the current burden of SABA use among patients with asthma as part of the SABINA program [4]. They reported that the prevalence of SABA overuse, defined as ≥ 3 inhalers per year, was 16% in Germany [4]. In our study, the prevalence was 36% in patients seen by GPs and 38% in patients seen by PNs. The main difference in the methodology between the study by Janson et al. and the present study was the required observation time. In the study by Janson et al., patients had to be followed-up for at least 12 months before and after study entry, whereas in our study, patients had to fulfill the observability criterion of having had at least two visits with their physician during the study period. Moreover, the SABINA study included patients treated by GPs only, and the present study additionally contained data of patients treated by PNs.

Our findings are in line with the results of other studies using the same definition of SABA overuse. In a large study from Poland based on pharmacy prescription records of more than 90,000 adult patients, SABA overuse was observed in 29–37% of patients [21]. In another study including approximately 16,000 patients in France, 28% of patients with asthma overused SABA therapy [22].

### Variables associated with SABA overuse

The proportion of patients with SABA overuse was higher in patients at GINA steps 3–5 (patients with probable severe asthma) compared with those at GINA step 1 or 2 (patients with probable mild asthma), showing poor asthma control in patients with severe asthma. This poor control was observed despite patients receiving ICS/LABA. At GINA steps 3–5, the risk of SABA overuse/increased use was even higher in patients using ICS/LABA. Patients treated by GPs were at a higher risk of overusing SABA compared with those treated by PNs. This finding could be attributed to non-familiarity of GPs with the updated GINA recommendations, which could have resulted in continued high prescribing of SABA [23]. Educational initiatives targeting physicians, pharmacists, and patients are required to align clinical practices with current treatment recommendations. PNs are more likely to prescribe SABA in line with therapy guidelines compared with GPs, given the PNs’ greater experience with asthma treatment. However, PNs may tend to treat more severe cases, which require earlier ICS/LABA and more SABA therapy.

In this study, SABA overuse was higher in men than in women. Several studies have investigated gender differences in asthma diagnosis and severity. Although female sex was shown to be an independent risk factor for severe asthma exacerbation among adults, [24], men exhibited lower therapeutic adherence with asthma therapy than women [24–26]. Our results are in line with the findings of Tavakoli et al. who reported that male sex was associated with a 1.49-fold higher likelihood of inappropriate SABA use [13].

### Consequences of SABA overuse

Although SABA are as-needed inhaled medications, they often appear to be used as long-term medications for asthma control, even though this is not in accordance with the guidelines [3]. Different complications of SABA overuse have been described in older and newer studies. A long-term study in New Zealand showed that regular use of SABA four times a day was associated with a deterioration in asthma control [27]. In a large Swedish population-based study, also part of the SABINA II program, which included data of more than 365,000 patients, SABA overuse was associated with an increased risk of asthma exacerbation and mortality [11]. The Swedish researchers used the same SABA overuse definition, that is, the use of ≥ 3 SABA inhalers within a 1-year period [11]. In a real-world, cross-sectional observational study by Azzi et al. SABA overusers were more likely to have moderate to severe nasal symptoms, tachycardia, vasodilation, transient hypoxemia, hyperglycemia, hypokalemia, and tremor [28]; a diagnosis of depression [5]; and an increased risk of emergency department visit or hospitalization [29, 30].

### Clinical implications

Our findings indicate that a high proportion of patients with asthma overuse SABA in Germany. Based on similar studies, this overuse probably carries a risk of adverse outcomes. There is no valid information on the reasons for the overuse of SABA. Both patients and physicians can have an impact on this. Physicians should eliminate SABA monotherapy and explain the consequences of SABA overuse to patients. Further, physicians, especially GPs, should ensure adherence of their patients to ICS treatment, considering continued ICS therapy can reduce the overuse of SABA. Asthma education programs for patients should pay special attention to regulated SABA use. Pharmacists play an important role in the care of patients with asthma and they should explain the appropriate use of SABA to patients who collect such medication from pharmacies.

### Strengths and limitations

The strengths of this study include the number of outpatients available for analysis and the use of real-world data, which allowed an unbiased exposure assessment (no recall bias) in a German cohort of patients with asthma for the first time. Moreover, we used a standardized definition of a SABA canister (~ 200 puffs/canister) to enable a more exact estimation of SABA overuse.

Retrospective primary care database analyses, however, are generally limited by the validity and completeness of the data they contain. First, diagnoses and comorbidities relied solely on ICD codes used in general physician and pneumologist practices, and no information was available regarding the procedure used to diagnose asthma. Asthma severity stages were established using GINA treatment steps according to the prescriptions of asthma medications because no documented information on the diagnosis of asthma severity or the level of asthma control was available; however, it is uncertain if physicians treated patients in line with these recommendations.

Second, data on the socioeconomic status (education and income) and lifestyle-related risk factors (smoking, alcohol use, and physical activity) of patients were lacking. Third, information from hospitals was not available. Fourth, receiving a prescription does not necessarily mean that the prescription was filled and used by the patient. There is a possibility that patients who received a prescription did not take the medication. In addition, information on whether patients received asthma education or written asthma action plans and their level of adherence to prescribed therapies was not captured in the study. Fifth, in the German healthcare system, a patient can receive prescriptions for asthma treatment from several doctors, for example, from both a GP and a PN; however, in this database, usually only one doctor’s prescriptions are captured. Sixth, no information on patients’ asthma training was available. Seventh, no separation of ICS/LABA therapy into reliever and controller was possible. Finally, the study was conducted in Germany, and its findings may not be extrapolated to populations in other countries due to differences in national health systems and the availability of SABA medications.

## Conclusions

In Germany, similar to other countries in the SABINA program, SABA overuse is prevalent among patients with asthma across all GINA steps, which may indicate suboptimal asthma control, suboptimal treatment practice, and suboptimal adherence to prescribed medication. Although, some original studies [4, 11], and a review article [31] have dealt with the problem of SABA overuse, further studies are needed to investigate the reasons behind the overuse of SABA.

## Data Availability

The datasets generated during and/or analyzed during the current study are not publicly available due to data protection rules but are available from the corresponding author on reasonable request.

## Abbreviations

ATC: Anatomical Therapeutic Chemical
EphMRA: European Pharmaceutical Market Research Association
GINA: Global Initiative for Asthma
GP: General practitioner
ICD-10: International Classification of Diseases, 10th Revision
ICS: Inhaled corticosteroid
LABA: Long-acting beta agonist
PN: Pneumologist
SABA: Short-acting beta-2 agonist
SABINA: **SAB**A use **IN A**sthma
